# Patterns of Treatment Interruption among Patients with Multidrug-Resistant TB (MDR TB) and Association with Interim and Final Treatment Outcomes

**DOI:** 10.1371/journal.pone.0070064

**Published:** 2013-07-29

**Authors:** Laura Jean Podewils, Maria Tarcela S. Gler, Maria Imelda Quelapio, Michael P. Chen

**Affiliations:** 1 U.S. Centers for Disease Control and Prevention, Atlanta, Georgia, United States of America; 2 Tropical Disease Foundation, Manila, Philippines; University of California, San Francisco, United States of America

## Abstract

**Background:**

The reasons that patients with multidrug-resistant tuberculosis (MDR TB) miss treatment are multi-factorial and complex. Identifying patterns of treatment interruption that predict poor outcomes can be used to target program activities aiming to improve treatment adherence.

**Objective:**

To characterize patterns of treatment interruption among MDR TB patients and determine the association between patterns and treatment outcomes.

**Methods:**

Retrospective analysis of MDR TB patients. A treatment interruption was defined as any time that a patient missed a prescribed dose of treatment for at least 1 day but for a period of less than 2 consecutive months. Patients were characterized by the number, length and variability of interruptions, variability of time between interruptions, and percent of missed doses. Final treatment outcome was dichotomized as a successful (cured or completed) or poor outcome (defaulted, failed, or died). Risk ratios were calculated to determine the association between characteristics of treatment interruption and treatment outcomes. All analyses were conducted in 6 month treatment intervals.

**Results:**

Only 7.0% of 583 patients completed treatment without interruption. Of the remaining 542 patients, the median time to the first interruption was 2 ½ months (70 days). In multivariate analysis, patients who had longer interruptions with sporadic variability during the 6–12 month or the 12–18 month treatment period had a significantly increased risk for poor outcomes compared to patients who had short, regular interruptions (RR_adj_ 4.37, 95% CI 1.2–15.8;  = 0.03 and RR_adj_ 3.38, 95% CI 1.6–7.1; p = 0.001, respectively). In addition, missing 10% or more of the prescribed doses during any 6 month period in the initial 18 months of therapy significantly increased the risk for poor outcomes (RR_adj_ range 1.55–2.35; p-value range 0.01–0.005).

**Conclusion:**

Patients that miss more consecutive days of treatment with sporadic interruption patterns or a greater proportion of treatment are at an increased risk for poor treatment outcomes.

## Introduction

In the Philippines, over 89% of patients with drug-susceptible smear positive tuberculosis (TB) complete their therapy or are successfully treated [1]. However, rates of successful cure or treatment completion among patients with drug-resistance or multidrug-resistant TB (MDR TB) are lower, estimated at 60% in 2006 [2]. Since MDR TB treatment is long and involves drugs with many adverse effects, default and treatment interruption is a common problem of programs. Patients that fail or default treatment have an increased risk for mortality, acquisition of additional drug-resistance, and promote continued transmission of drug-resistant *Mycobacterium tuberculosis (M. tuberculosis)* strains in the community.

Studies in drug-susceptible TB patients have suggested that treatment interruptions are associated with an increased risk for treatment default, failure, or death [3]–[5], but the characteristics and patterns of missed doses and treatment interruptions that are most predictive has not been well established in patients with MDR TB. Different patient groups may also be associated with higher probability of interrupting treatment [6]–[8]. Further, the methods used to define treatment interruption have not been standard across studies with some defining interruption as any single missed treatment dose [4], [5], [7], utilizing the number of times treatment was interrupted [4], the median or total duration of missed doses [5], [6], maximum number of consecutive doses missed [4], [7], or cut-offs for proportion of total treatment that was missed [6], [8]. It is possible that some crude definitions lack sensitivity to identify patients at greatest risk for poor outcomes; other categorizations may have differing relevance to outcomes within a program context.

If certain patterns of treatment irregularity identify MDR TB patients at highest risk for treatment default, failure, or death, targeting interventions to these patients earlier in their treatment course may help to optimize treatment adherence and ultimately improve treatment outcomes.

The objectives of the present study are to 1) expand the simple definitions of treatment interruption by considering a more complete set of measures that characterize patterns of missed doses (treatment interruptions) among MDR TB patients, 2) identify patterns from these measures, and 3) determine the association between patterns and bacteriologic and microbiologic parameters over the course of and at the end of MDR TB treatment.

## Materials and Methods

### Study Population

A retrospective study was conducted among all patients with MDR TB enrolled in the Phillipine Program for the Management of Drug-Resistant TB (PMDT) between 15 April 1999 and 31 December 2006 [9]. The present nested cohort analysis included all MDR TB patients managed at the Makati Medical Center (MMC) Directly Observed Therapy, Short-course (DOTS) Clinic who had treatment cards available and a known treatment outcome.

### Program Description

The MMC DOTS Clinic is an outpatient MDR TB clinic that provides directly observed therapy (DOT) by a health worker 6 days per week throughout the treatment period. Treatment regimens included at least 4 drugs to which the patient’s isolate was susceptible, including a second-line injectable drug (amikacin, kanamycin, capreomycin), a fluoroquinolone (ciprofloxacin, ofloxacin, levofloxacin), and at least two of the group 4 or 5 drugs (cycloserine, prothionamide, paraaminosalysilic acid, clarithromycin) during the intensive phase (first 6 months or 156 doses after culture conversion). After completing the intensive phase of treatment, the patient is shifted to the continuation phase of treatment during which the injectable agent is discontinued for the remaining 12 months, or 312 doses, of the treatment course. Patients are eligible for decentralization, where TB treatment and management are transferred to a TB DOT clinic close to the patient’s residence, following culture conversion. When a patient interrupts treatment for 2 consecutive days, health workers try to contact the patient by telephone; patients are visited at their residence if they interrupt treatment for more than 6 days. Each contact involves reorientation on the importance of treatment adherence.

### Patient and Treatment Characteristics

Sociodemographic factors and other patient characteristics including geographic location, body mass index, comorbidities, history of smoking, alcoholism, substance abuse, number of previous treatments, history of default from previous anti-TB treatment, and extent of disease were collected from patient records. Information on drug-sensitivity results for first and second-line anti-TB drugs, drugs used in the treatment regimen and adverse drug reactions was also collected. All of these variables are recorded as part of the national standard of MDR TB patient care. Social and medical history variables were categorized as either having or not having a known history.

#### Treatment interruptions/missed doses

A treatment interruption was defined as any time that a patient missed a prescribed dose of MDR TB treatment for at least 1 day but for a period of less than 2 consecutive months. Using MDR TB Category IV treatment cards, data on whether or not a patient took the dose for each day of treatment was abstracted by a trained data abstractor and recorded onto standardized forms designed for the study. Each patient therefore contributed a trajectory of time for the entire treatment course, with dates demarking time periods for when the patient missed doses (treatment interruptions) and those when the patient was on treatment between interruptions (time on treatment).

#### Interim treatment indicator: 6 month culture conversion

Patients with a negative culture for *Mycobacterium tuberculosis* at the end of 6 months of MDR TB treatment with 2 subsequent months of negative cultures were established as having culture conversion at 6 months.

#### Final mdr TB treatment outcomes

Final MDR TB treatment outcomes were based on international consensus guidelines distinguishing the following categories: cure, completion, default, failure, and death [10], [11]. In brief, an outcome of treatment cure was based on bacteriologic culture demonstrating at least five consecutive monthly cultures in the last 12 months of treatment or having completed 468 doses after culture conversion, or a single positive culture during this time followed by at least three negative monthly cultures. Treatment completion was assigned to patients who completed the required number of treatment doses but did not meet the criteria for confirming treatment cure. An outcome of default refers to patients who interrupted treatment for ≥2 months (8 weeks) consecutively without a return to treatment. Patients were considered treatment failures if they had more than one positive culture in the final 12 months of therapy or otherwise terminated treatment early.

### Statistical Analysis

#### Treatment interruption measures

Several different patterns and categorizations of missed doses were derived and calculated from the data. The total number of treatment interruptions and the total proportion of missed doses (total days of doses missed/total days on treatment X 100) were calculated for each patient. The mean and standard deviation for each the duration of treatment interruption and the duration of time on treatment between interruptions was also calculated for each patient. Recognizing that treatment adherence patterns may vary over the 18–24 month treatment period, each metric characterizing treatment adherence patterns was calculated and evaluated for the entire treatment course and for each 6 month treatment interval.

**Figure 1:**
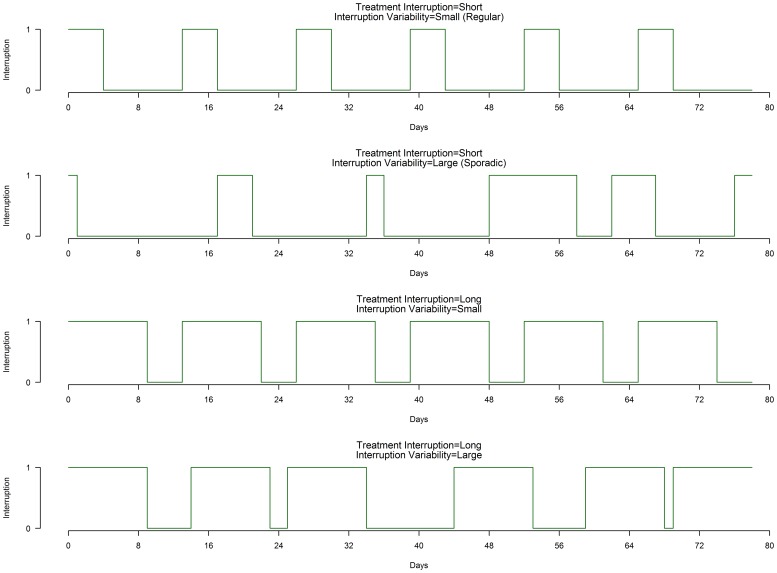
Illustration of 4 different treatment interruption categories, based on length of interruption in days (short or long), and variability of interruption (small/regular or long/sporadic). Y-axis indicates treatment interruption as 0 (no; on treatment) or 1 (yes; interruption/missed dose).

To categorize distinct patterns of treatment interruption, two dichotomous variables were created using the median (across all patients that had missed a dose during the time period) as a cut-point for each the individual mean length of interruption (duration) and the individual standard deviation (variability) of treatment interruption. These 2 variables were then combined to create 4 distinct treatment interruption patterns based on the characteristics (duration and variability) of the treatment interruption (where short or small is less than the median and long or large is greater than the median): 1) short mean treatment interruptions, small variability (regular); 2) short mean treatment interruptions, large variability (sporadic); 3) large mean treatment interruptions, regular; and 4) large mean treatment interruptions, sporadic (Figure 1).

#### Interim and final treatment outcomes

Culture conversion at 6 months was used as the dependent variable for interim treatment outcome. Final treatment outcome was dichotomized as a successful or poor outcome, with success including patients that were cured or completed treatment and a poor outcome comprised of patients that defaulted, failed, or died.

#### Data analysis

The goal of this analysis was to evaluate treatment adherence among MDR TB patients in different periods of treatment, as implementation of intervention activities in earlier phases of treatment may be critical to optimizing treatment outcomes. Thus, the analysis focused on the details of interruption patterns in each 6 month treatment period. The analysis involved three components: 1) describing and characterizing patterns of treatment interruptions; 2) among patients with at least one treatment interruption, evaluating the association between different treatment interruption patterns and a) interim outcomes (culture conversion) and b) final treatment outcomes; and 3) describing the patient characteristics associated with category of interruption pattern.

Descriptive statistics were used to describe characteristics of treatment interruptions. The frequency of pattern changes was used to evaluate the consistency of interruption patterns across 6 month treatment time periods for each individual. Rate ratios (RR) and 95 percent confidence intervals (CI) were calculated to quantify: a) the association between treatment interruption pattern characteristics and interim and final treatment outcomes, and b) the association between patient characteristics and treatment interruption patterns previously identified as associated with the final treatment outcome (as a nominal outcome variable). Only the treatment patterns during the first 6 month interval were considered to evaluate the association with the interim outcome of smear conversion. Patient sociodemographic and baseline clinical factors and factors related to the treatment course were considered as covariates in models evaluating the association between interruption characteristics and interim and final treatment outcomes. All multivariate models that included treatment interruption patterns adjusted for time on treatment variability. Initially all characteristics were evaluated using univariate models for each 6 month period (0–6, 6–12, 12–18, and 18–24 months), and multivariate Poisson regression models were constructed considering variables that had a p-value ≤0.20 in univariate models, biologic plausibility, or had been previously demonstrated to be important based on previous research. Each Poisson model included robust estimators of the variance, and the deviance was calculated for each model to assess goodness of fit. All data analyses were conducted using Stata version 12.0 (College Station, Texas, USA). An alpha level <0.05 was considered statistically significant.

### Ethical Review

The study was reviewed and approved by the Tropical Disease Foundation Institutional Review Board (IRB). Informed consent was waived because the study was limited to review of medical records and was therefore considered to be minimal risk. IRB review at the U.S.

Centers for Disease Control and Prevention (CDC) was not required because CDC investigators did not interact with patients or have access to identifiable patient information.

## Results

### Study Population

Of the 583 MDR TB patients, the median age was 37.5 (IQR 28.9–48.9) and the majority of patients were male (60.2%) (Table 1). Almost half (48.5%) of patients were underweight, and over one-third were current smokers or had a history of alcohol abuse. Most patients had pulmonary TB, and 86.3% had at least two previous episodes of TB treatment prior to the current MDR TB diagnosis. The majority (n = 558; 95.7%) of patients were culture-confirmed and had DST results; 25 (4.3%) patients were treated empirically. Over two-thirds (68.4%) of patients were cured or completed treatment (n = 399; 384 cured +15 completed). Among patients with poor treatment outcomes, default was the most common (n = 88; 15.1% of all patients); 12.9% of patients died during the treatment course (n = 75), and 3.6% failed treatment (n = 21).

**Table 1:** Sociodemographic characteristics of MDR TB patients included in the present analysis (n = 583).

Characteristic	n (%)			
**Age, years (median, IQR)**	37.5 (28.9–48.9)			
**Sex**				
** Female**	232	(39.8)		
** Male**	351	(60.2)		
**Marital Status**				
** Married**	352	(60.5)		
** Single/Divorced/Widowed**	230	(39.5)		
**Employment Status**				
** Employed**	191	(34.1)		
** Unemployed/Student/Retired**	370	(66.0)		
**Area of Residence**				
** National Capital Region**	386	(66.2)		
** Other**	97 (33.8)			
**Income Level, PHP/month** *				
** <5000**	131	(22.5)		
** ≥5000**	452	(77.5)		
**Body Mass Index (kg/m^2^)**				
** Underweight (<18.5)**	249	(48.5)		
** Normal**	264	(51.5)		
**History of Diabetes**				
** No**	402	(73.6)		
** Yes**	144	(26.4)		
**Current Smoker**				
** No**	343	(58.8)		
** Yes**	240	(41.2)		
**History of Alcohol Abuse**				
** No**	331	(56.8)		
** Yes**	252	(43.2)		
**Site of TB Disease**				
** Pulmonary**	569	(97.6)		
** Extrapulmonary**	14	(2.4)		
**Previous TB treatment episodes**				
** None**	12	(2.1)		
** 1**	68 (11.7)			
** ≥2**	503	(86.3)		
**Cavitation at diagnosis**				
** No**	272	(46.7)		
** Yes**	311	(53.3)		
**Drug Susceptibility Results** †				
**1^st^ line only**	350 (62.7)			
**1^st^ line +2^nd^ line injectable only**	2 (0.3)			
**1^st^ line+fluoroquinolone only**	186 (33.3)			
**1^st^ line+fluoroquinolone+injectable** **(XDR TB)**	22 (3.9)			
**Drugs Used in Treatment Regimen** §				
***First-line***				
**Pyrazinamide**	352 (60.5)			
**Ethambutol**	83 (14.2)			
***Injectable Drugs***				
**Kanamycin or Amikacin**	385 (66.0)			
**Capreomycin**	38 (6.5)			
**Streptomycin**	203 (34.8)			
***Fluoroquinolones***				
**Ciprofloxacin**	17 (2.9)			
**Ofloxacin**	284 (48.7)			
**Levothoxacin**	26 (4.5)			
**Moxifloxacin**	185 (31.7)			
***Group 4 or 5 Drugs***				
**Clarithromycin**	47 (8.1)			
**Cycloserine**	505 (86.6)			
**Prothionamide or Ethionamide**	425 (72.9)			
***p-*** **aminosalicylic acid**	320 (54.9)			

XDR TB = extensively drug-resistance tuberculosis.

* 5000 PHP/month equals approximately $120 US dollars/month.

† DST missing for 25 patients who were treated empirically (n = 558 with DST). DST for 1^st^ line drugs included isoniazid (H), rifampin (R), pyrazinamide (Z), ethambutol (E); injectable drugs tested included streptomycin (S) and kanamycin (K); 2^nd^ line floroquinolones tested included ciprofloxacin (Cpx) and ofloxacin (Ofx).

§ Drugs are not mutually exclusive, and indicate number and proportion of patients who were prescribed the drug at some point during the treatment course.

Values represent number of patients and proportion unless otherwise noted.

### Treatment Interruptions

**Figure 2:**
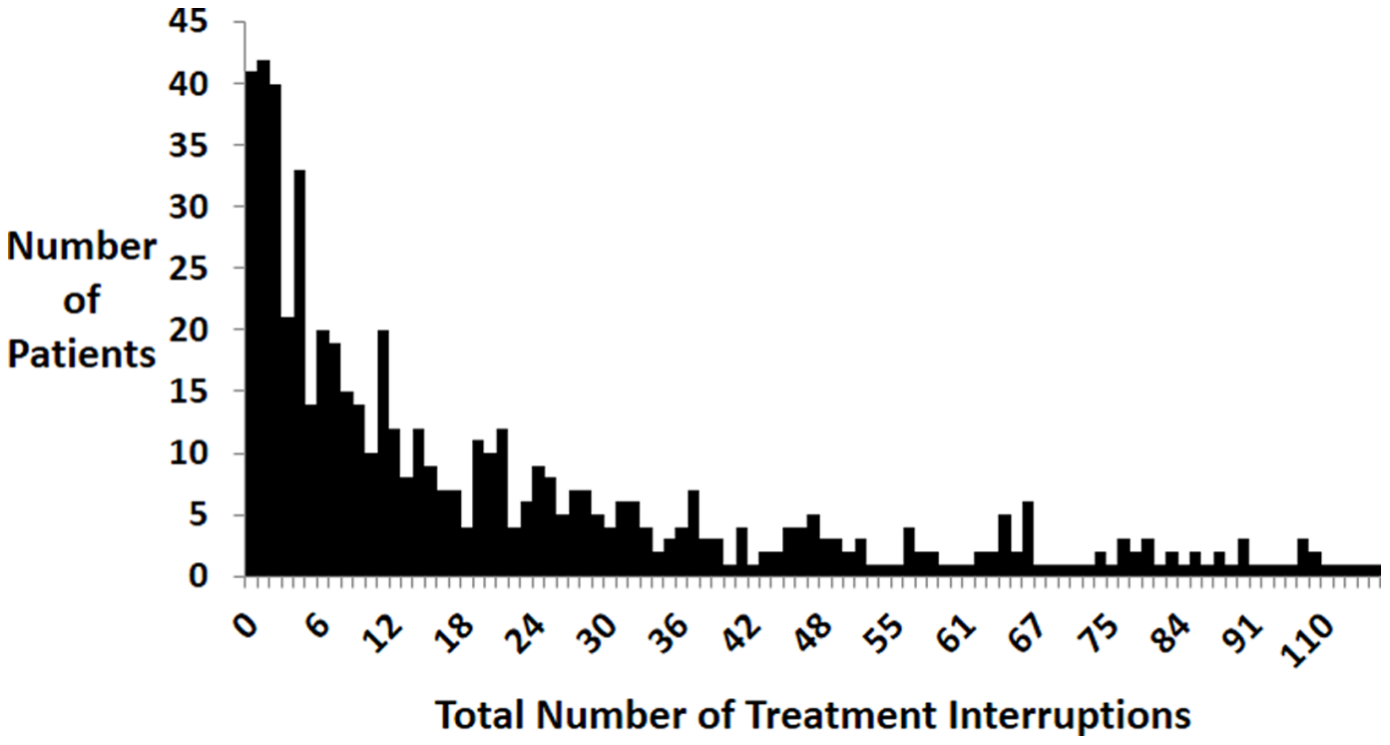
Distribution of number of total treatment interruptions among MDR TB patients, 1999–2006 (n = 583).

Only 41 (7.0%) patients did not miss any treatment during the treatment course (Figure 2). Of the 542 remaining patients, the number of interruptions ranged from 1 to 205 and the median number of interruptions was 14. The median length of time to the first interruption was around 2 ½ months, at 70 days (Table 2). Patients missed a median average (pooled median of individual mean time of interruption) of 1.4 days per interruption (range 1–37). Conversely, the median average length of time that a patient was on treatment before another missed dose, was just about one month (29.4 days). The total number of days missed over the treatment course had a median of 23 days (range = 1–446, IQR 7–63). The median proportion of total treatment duration that was missed was 4.5 percent (median range 3.8 to 9.7% from periods 0–6 through 18–24 months). One hundred and fifty (150/543; 27.7%) patients who had treatment interruptions missed ten percent or more of the total doses prescribed.

**Table 2:** Treatment interruption characteristics of MDR TB patients that missed at least one dose during the course of treatment (n = 542).

Characteristic	Median (IQR)	Range
**Time to first interruption,** **days**	69.5 (24.0–173.3)	1–610
**Duration of interruption,** **days** *	1.4 (1.1–2.0)	1–37
**Duration of treatment episode,** **days** *	29.4 (14.3–69.5)	2–345
**Total number of missed doses,** **days**	23 (7–63)	1–446
**Percentage total missed** **doses**	4.5 (1.8–11.4)	0.1–66

IQR, interquartile range.

* Characteristics measuring duration of interruptions and duration of treatment episodes were averaged within each patient; values in the table represent the median and IQR of these averages.

### Treatment Interruption Categories

Overall, 43.9% of patients with interruptions exhibited a pattern with long, sporadic interruptions (pattern 4; Table 3). This proportion was relatively consistent across 6 month treatment time categories. Almost a quarter of patients with interruptions had each short, regular interruption patterns or short, sporadic interruption patterns (23.7 and 23.2%, respectively). Evaluating consistency across time periods for each individual, 33.9% (184/542) of patients demonstrated consistent patterns across the treatment course ((n = 52 (9.6%) with an identical pattern for all 4 6 month time periods and n = 132 (24.4%) with matching patterns for 3 of the 4 6 month treatment intervals)). Patients with long, sporadic interruptions (pattern 4) were the most consistent ((n = 117 (21.6%) demonstrating pattern 4 for 3 or 4 of the time periods)).

**Table 3:** Treatment patterns among MDR TB patients, by 6 month intervals of treatment (n = 583).

Category Characteristics				Treatment Time								
	**Interruption** **Duration**		**Interruption** **Variability**		**0–6 mos.** **(n = 583)**	**6–12 mos.** **(n = 513)**			**12–18 mos.** **(n = 479)**	**18–24 mos.** **(n = 424)**	**Overall (0–24 mos.) (n = 583)**	
**1**	**Short**	**Small (Regular)**			0 (0.0)		40 (7.8)		73 (15.2)	25 (5.9)	138 (23.7)	
**2**	**Short**	**Large (Sporadic)**			200 (34.3)		160 (31.2)		133 (27.8)	101 (23.8)	135 (23.2)	
**3**	**Long**	**Regular**			0 (0.0)			0 (0.0)	1 (0.2)	2 (0.5)	12 (2.1)	
**4**	**Long**	**Sporadic**			213 (36.5)		218(42.5)		192 (40.1)	140 (33.0)	256 (43.9)	
	**No missed doses during period**				170 (29.2)	95 (18.5)			80 (16.7)	160 (37.4)	41 (7.0)	

Four categories of treatment interruption patterns based on the patients in the period that had at least one treatment interruption (missed dose) during that period. Cut-offs to create categories based on the median of interruption characteristics (individual mean interruption length in days and the individual standard deviation of the interruption length in days) during the period, wherein individual values below and inclusive of the median value denoted short or small/regular and values exceeding the median cut-off were categorized as long or large/sporadic. The pooled median of individual mean days of interruption and standard deviation of interruption used for cut-offs was 1.14, 0 for 0–6 months; 1.21, 0.38 for 6–12 months; 1.28, 0.50 for 12–18 months, and 1.33, 0.51 for 18–24 months. N in parentheses at the top of each column reflects number of patients who were still on treatment at the beginning of the 6 month period.

### Interim Outcomes: Association between Interruptions in Initial 6 Months of Treatment and Interim Outcomes

Information on culture conversion was missing for 9 patients; 88.7% (509/574) of patients with available laboratory information were culture negative at the 6 month follow-up. Pattern of treatment interruption in the initial 6 months of treatment was not significantly associated with failure to convert to a negative culture at 6 months (long, sporadic vs. short, sporadic RR = 1.55, 95% CI 0.9–2.8.0; p = 0.15). In a multivariate model adjusting for age and site of disease (pulmonary or extrapulmonary), patients who missed more than 10% of treatment doses in the first 6 months had a significantly higher risk of failing to convert to a negative culture at the 6 month follow-up (RR_adj_ = 2.47, 95% CI 1.4–4.4; p = 0.002).

### Final Treatment Outcomes

In multivariate analysis, patients who had longer length interruptions with sporadic variability during the 6–12 month or the 12–18 month treatment period had a significantly increased risk for poor outcomes compared to patients who had short, regular interruptions during the treatment course (RR_adj_ 4.37, 95% CI 1.2–15.8; p = 0.03 and RR_adj_ 3.38, 95% CI 1.6–7.1; p = 0.001, respectively) (Table 4). Poor outcomes were also more likely among patients with short, sporadic treatment interruption patterns during the 12–18 month period (RR_adj_ 2.54, 95% CI 1.1–5.7; p = 0.03). In addition, excepting the final 18–24 months of treatment, there was an independent and significant effect associated with missing a greater proportion of doses during the period, with a 1 ½ to 2-fold increase associated with missing 10% or more of prescribed treatment doses (RR_adj_ range 1.55–2.35; p-value range 0.01–0.005). All models in all periods were adjusted for age, number of interruptions during the period, and variability of time on treatment; each model also included variables identified as significantly associated with poor outcome, specifically being underweight (BMI<18; p-value range 0.002–0.02), receiving kanamycin or amikacin (p-value <0.001 for all treatment periods), or receiving clarithromycin (p-value range <0.001–0.05) during the treatment course. No other patient sociodemographic, clinical, or treatment characteristics, including previous TB treatment, drug-susceptibility profile, indicators of disease severity (e.g., cavitation), or adverse events were significantly associated with poor outcomes in univariate or multivariate models.

**Table 4:** Multivariate association between treatment interruption pattern characteristics for each 6 month treatment period and poor treatment outcomes (default, failure, death) among MDR TB patients with at least one missed dose during the course of treatment (n = 542).

	0–6 months		6–12 months		12–18 months		18–24 months	
Characteristic*	RR† (95% CI)	p	RR† (95% CI)	p	RR† (95% CI)	p	RR† (95% CI)	p
**Interruption Pattern**								
**Short, regular**	*reference*		*reference*		*reference*		*reference*	
**Short, sporadic**	–		3.32 (0.9–12.0)	0.07	**2.54 (1.1–5.7)**	**0.03**	0.77 (0.2–3.9)	0.75
**Long, regular**	–		–	–	–	–	1.0 (1.0–1.0)	1.0
**Long, sporadic**	1.10 (0.8–1.6)	0.61	**4.37 (1.2–15.8)**	**0.03**	**3.38 (1.6–7.1)**	**0.001**	2.78 (0.6–13.1)	0.20
**Missing >10% of Treatment Doses**	**1.55 (1.1–2.4)**	**0.05**	**1.97 (1.2–3.4)**	**0.01**	**2.35 (1.3–4.3)**	**0.005**	1.26 (0.5–3.0)	0.60

RR, rate ratio; CI, confidence interval.

* Each variable represents characteristic for the 6 month treatment period (e.g., 0–6 months).

† All RRs adjusted for age, number of treatment interruptions, variability of time on treatment, and whether they were underweight (BMI<18), or received kanamycin/amikacin or clarithromycin during the treatment period. RRs presented are adjusted for all other variables in the model. Dashes (–) for RR and 95% CI indicate there were not enough observations in the interruption category for that time period to derive an estimate.

### Association between Patient Characteristics and Treatment Patterns

Since the interruption pattern of long, sporadic interruptions was identified as independently associated with a significantly increased risk for poor treatment outcomes in more than one 6 month treatment period, had a consistent point estimate above 1 for all treatment periods, and was the most consistent within patients across all treatment periods, patterns were dichotomized as long, sporadic interruptions or other interruptions in each 6 month treatment period. Patients who were employed at the time of initiating MDR TB treatment were 22% more likely, if they interrupted treatment, to demonstrate long, sporadic interruption patterns during the 6–12 month period of TB treatment than other patterns, compared to patients who were unemployed (age-adjusted RR 1.22, 95% CI 1.0–1.5; p = 0.04). No other factors were associated with the long, sporadic interruption pattern in the 6–12 month treatment period. No sociodemographic or baseline clinical factors were significantly associated with the long, sporadic interruption pattern in the 0–6, 12–18 month or 18–24 month treatment periods.

When evaluating the association between patient characteristics and proportion of missed doses, proportion of missed doses was categorized using a cut-off of 10% for the given treatment period. In a multivariate model also adjusting for age, patients with extrapulmonary disease or both pulmonary and extrapulmonary TB (vs. pulmonary TB only; RR_adj_ 2.9, 95% CI 2.4–3.5; p<0.001) or with a history of alcohol abuse (RR_adj_ 1.51, 95% CI 1.2–2.0; p = 0.003) were more likely to miss 10% or more of doses during the 6–12 month treatment period. Patients with a history of alcohol abuse were also more likely to miss a greater proportion of treatment doses during the 12–18 month and the 18–24 month treatment periods (12–18 month age-adjusted RR 1.37, 95% CI 1.1–1.7, p = 0.01; 18–24 month age-adjusted RR 1.31, 95% CI 1.1–1.7, p = 0.03). There were no sociodemographic or clinical factors associated with the proportion of doses missed during the initial 0–6 months of treatment.

## Discussion

In the present study, only a small proportion (7.0%) of patients with MDR TB were able to completely adhere with treatment over the entire treatment course. However, the treatment interruption behaviors for these patients vary widely. This study demonstrates that patients on MDR TB treatment are bound to interrupt or miss their doses and certain patterns may lead to poor outcomes. Patients who interrupt their treatment for longer periods with sporadic intervals, particularly between 6 and 12 months and 12 to 18 months after initiating treatment, have 3–4 times the risk for poor outcomes. Though the association between the long period with sporadic interval treatment interruption category and poor outcome were not significant for the initial or the final 6 months of treatment, the point estimates were above 1, and lack of significance may be a result of fewer interruptions or fewer patients still on treatment. Our results also indicate that the change of patterns from one six month period to another is infrequent. Sporadic interruptions with shorter duration during the 12–18 month period were also significantly associated with poor outcomes; it is possible that the variability of missing treatment may signal another factor that may directly or indirectly interfere with treatment effectiveness. Missing 10% or more of treatment doses during the first 18 months of treatment was also associated with an increased risk for poor outcomes. Treatment interruptions with sporadic intervals or missing a greater total of prescribed treatment doses may inhibit the capacity to achieve or maintain a therapeutic drug level necessary to effectively treat MDR TB.

It is possible that MDR TB patients begin to feel better with resolution of symptoms such as cough and fever after the initial phase of treatment and may therefore be more likely to interrupt treatment, as has been cited in previous studies among drug-susceptible TB patients [12]. Interruptions during the 6–12 and 12–18 month periods of MDR TB treatment may be particularly critical, when the patient is expressing clinical improvement but the *M. tuberculosis* has not been fully cleared, potentially facilitating bacterial replication and acquisition of additional drug-resistance. Early detection of these types of interruptions may signal programs to use alternative strategies to default tracing and patient education, including home or workplace DOT. A recent study has shown the cost-effectiveness of ambulatory MDR TB care [13]; however, a single-model, clinic– based treatment may not be the only answer.

To our knowledge, this is the first study examining treatment interruptions among patients with MDR TB. Previous studies in drug-susceptible TB patients have used various simple indicators for categorizing treatment interruption, including the presence or absence of a missed dose during treatment. These studies have shown that the presence of treatment interruption during the intensive phase was associated with poor outcome [3], [5]. In MDR TB, categorizing patients solely based on the proportion of missed doses during the intensive phase or continuation phase may not adequately identify patients at risk for poor treatment outcomes, as we identified an independent effect of particular treatment patterns on outcome. We divided the treatment course into 6-month intervals, to better understand the association of different adherence characteristics during each stage of the treatment course to final treatment outcomes, and as an approach that may be useful to program managers. It is important to examine interruptions among MDR TB patients intricately and routinely in order to effectively target interventions, particularly in the context of limited resources.

We were unable to identify any significant association between treatment interruption pattern in the initial 6 months of MDR TB therapy and culture conversion at the end of the intensive phase of treatment. While we cannot discount this finding as a true lack of association, it is possible that the null associative value was due to the majority of patients achieving conversion at this time. However, consistent with the finding of an increased risk for a poor outcome among patients who missed at least 10% of their treatment during any of the first three 6-month treatment periods, patients who missed 10% or more of doses in the first 6 months of TB drug therapy also were at risk for not converting to a negative culture at the 6-month follow-up.

Of interest, our findings suggest that employment may contribute to longer and variable interruptions in treatment. Other studies have cited work commitments as a key barrier to TB treatment adherence [14], [15]. Programs may need to consider implementing alternative strategies (e.g., workplace DOT) to administer daily treatment for patients who are employed, and interventions may include increasing knowledge about the importance of TB treatment among employers. In addition, patients with extrapulmonary disease or a history of alcohol abuse were more likely to miss a greater proportion of treatment later in the treatment course. When these variables were considered in the models evaluating patterns and final treatment outcomes they did not retain independent associations with treatment outcome. It is possible that treatment interruptions represent an intermediate mechanism that more closely predicts the effectiveness of treatment, and employment status and alcohol abuse may not directly influence outcome excepting their impact on treatment adherence. This finding underscores the importance of monitoring and summarizing treatment interruptions among all patients, and suggests a need for close scrutiny to patients with factors that may inhibit their ability to be adherent.

As with any study, ours is not without limitations. Our focus was on identifying intermediate markers that may lend themselves to program interventions to optimize treatment outcomes; while an overall adoption of program strategies to assess treatment interruption patterns and target interventions accordingly may improve outcomes, its impact on decreasing the proportion of patients who default or die early may be less successful. The current study did not collect information on patient related or programmatic reasons that may contribute to missing doses of TB treatment. Previous research identified that inadequate training and supervision of health care staff responsible for TB management and DOT were significantly associated with interrupting treatment, and patients with a poor understanding of reasons to stop treatment missed more days of treatment [7]. Patient lack of understanding about disease severity also has been demonstrated to increase risk for interrupting treatment [6]. In this study, patients who were employed at the time of treatment initiation were more likely to demonstrate interruption pattern characteristics associated with an increased risk for poor outcomes. However, we only measured patient sociodemographic and clinical characteristics at baseline, and therefore cannot conclude whether the observed association may be due to work-related constraints or whether these individuals may have suffered a loss of employment over the treatment course, thereby incurring additional financial and other practical barriers to adherence [16]. Finally, the relationship between different interruption patterns and treatment outcomes observed in our study may differ from that in other programs or settings. We examined the characteristics of treatment interruptions among a large cohort of patients with MDR TB who were given DOT throughout the treatment duration, allowing for detailed characterization of treatment interruptions and evaluation of how different treatment patterns influenced final treatment outcomes. Our analysis revealed the dynamics of treatment adherence at the individual patient level from the beginning to end of the whole MDR TB treatment period, and identified risk factors associated with treatment interruptions in each 6-month period. These practical intermediate results would allow programs to employ interventions aimed at improving treatment adherence earlier in the treatment course. The novel approach and methods used in this analysis may be useful to other programs aiming to identify modifiable factors during the treatment course that may influence treatment outcomes.

Based on our findings, we believe that identifying these high-risk patients through careful and routine review of treatment cards will help target program interventions to improve adherence and treatment outcomes. Awareness of such patterns could be a signal for programs to use strategies beyond default tracing such as home DOT or workplace DOT. Future research should aim to identify underlying reasons that contribute to different patterns of treatment interruption to guide and optimize the effectiveness of program activities.

### Disclaimer

The findings and conclusions in this report are those of the authors and do not necessarily represent the views of U.S. Centers for Disease Control and Prevention.
